# The Applicability of N: Ancient Debates and Modern Experimental Design

**DOI:** 10.3390/healthcare6030118

**Published:** 2018-09-19

**Authors:** Katherine D. van Schaik

**Affiliations:** Harvard Department of the Classics, Harvard Medical School, Boston, MA 02115, USA; k.d.van.schaik@gmail.com

**Keywords:** medical humanities, N-of-1 trials, clinical trials, history of medicine

## Abstract

Medicine has always been characterized by a tension between the particular and the general. A clinician is obligated to treat the individual in front of her, yet she accomplishes this task by applying generalized knowledge that describes an abstract average but not necessarily a specific person. Efforts to systematize this process of moving between the particular and the general have led to the development of randomized controlled trials and large observational studies. Inclusion of tens of thousands of people in such studies, it is argued, will enhance the applicability of the data to more individual circumstances. Yet, as genetic sequencing data have become more widely obtained and used, there has been an increased focus on what has been broadly termed “precision medicine”, a highly individualized approach to therapeutics. Moreover, advances in statistical methods have enabled researchers to use N-of-1 study data—traditionally considered too individualized to be broadly applicable—in new ways. This paper contextualizes these apparently modern debates with reference to historical arguments about methods of disease diagnosis and treatment, and earlier physicians’ concerns about the tension between the particular and the general that is intrinsic to medical practice.

## 1. Ancient Debates: Individualities or Generalities?

In the Hippocratic treatise *Places in Man*, written around the mid-fifth to the fourth century BCE in classical Greece, the author explains the challenges of developing a framework for medical theory and practice. Focusing on navigating the unpredictable terrain between general principles and individual variability, the author cautions his pupils (*Places in Man* 49):

Medicine cannot be learned quickly, because it is impossible for there to exist any established method in it, as for example when someone who has learned to write in one way that is taught then understands everything. Medicine from one moment to the next does things that are opposite, and it does opposite things for the same person, indeed, even things that are self-contradictory.

This is an early articulation of a dilemma that would preoccupy physicians for the centuries that would follow. In the Greco-Roman world in particular, schools of medical thought and practice differed in their conceptualization of the cognitive processes associated with the clinical encounter: should a physician proceed on the basis of patients’ differences, or on the basis of similarities? How generalizable was a physician’s knowledge? How many patients did a physician need to see, or how many occurrences of a given pathology did he need to treat, before he felt confident in his ability to identify or to remedy that problem in a new patient or a different context?

The diversity of thought, the variety of practice, and the depth of epistemological debate that characterized the art of medicine in Greco-Roman antiquity are all too great to summarize adequately in one short section of a brief article, and this article does not claim to do so. Nutton [1] has compiled an accessible, comprehensive, and readable overview of these debates, and other scholars have described the evolving philosophical milieu from which these discussions emerged [2,3,4,5,6]. This section will mention certain elements of the ancient discussion around the tension between the particular and the general as they relate to contemporary arguments about experimental design and the development and applicability of modern therapeutics.

Although there were many schools of medical thought and practice in the ancient Mediterranean world, three have been special subjects of in-depth scholarly discussion in the 20th and 21st centuries. These are the Empiricists, the Rationalists, and the Methodists. In brief, Empiricists were so named from their focus on *empeiria*, “experience,” in medical training, diagnosis, prognosis, and treatment. They relied on an epistemological “tripod” of (1) the reliably-recorded experience of other physicians (“history”), (2) a practitioner’s personal intellectual store of cases in which he has been involved, and (3) a method of reasoning called “the transition to the similar” [7]. This third intellectual tool enabled an Empiricist physician to make clinical management decisions about an unfamiliar scenario by identifying features of that case that were similar to those of cases he had previously encountered. The more cases a practitioner had read about or, better yet, had managed himself, the better physician he would be. His understanding of the theoretical physiological mechanisms behind disease, or of the theoretical justifications for the remedy he might suggest, was less important than his personal “N”—the number of different cases he had seen and treated.

Rationalist physicians, so titled by later scholars, were more concerned than Empiricists and Methodists with the theoretical mechanisms behind patients’ pathologies and their recommended remedies. The physician Galen (129-c210CE), born in modern-day Turkey and ultimately the doctor to the Roman imperial family, draws much of his approach from the Rationalist school of thought (although his focus on experience treating patients as a cornerstone of medical training shows the influence of Empiricist doctrine). Galen was interested in the physiology he could not directly observe, and his development and articulation of anatomically-focused, humoral medicine—and his assertion that each individual had a unique ‘normal’ that could become deranged in particular ways—became the foundation for medieval European and Islamic medicine. His words endured as the standard theoretical model and daily practice up to the 17th century.

Methodist physicians, named after their *methodos* (“method”), were committed to a view of medical theory and practice rooted in three so-called “commonalities” into which any patient’s clinical presentation could be classified: stricture, laxity, or a mixed state. They recognized that these three over-arching commonalities could manifest in different parts of the body, in different ways, states they referred to as *pathē*. They believed that Empiricist and Rationalist physicians had over-complicated medical practice, and that their emphasis on seeing a large number of patients, or on theoretical justification for their therapies or prognoses, was not useful in the immediate diagnosis and treatment of patients. The Methodist approach involved acknowledgement of the similarities across disease states and recognition of the consistency in human anatomy and physiology.

A point of contention between Galen and his Methodist rivals involved, in Galen’s words, their treatment of “the common man.” [8] As described above, their system focused on the similarities among patients. Unlike Galen, they were less convinced that a practitioner needed to determine or to characterize a patient’s unique nature, diet, habits, or exposures in order to treat that patient’s problem. They observed the patient’s symptoms, identified the commonality that was responsible, and then proceeded with the treatment that was indicated by the commonality. Generalities helped them to practice medicine efficiently and systematically, and contemporary scholars have argued that the Methodists’ generalized approach to medical treatment would have been useful in crowded urban hospitals [Celsus, *On Medicine*, Proem 64–65], and was potentially preferred by patients, since it favored regular patient visits every two to three days, until the end of the illness [9].

In the centuries that followed the comment made by the Hippocratic writer of *Places in Man*, the medical practitioners of Greco-Roman antiquity developed new ways to navigate between the individual patient and more generalized conceptions of disease, treatment, and health. Yet, even though modern clinicians are able to diagnose and to treat with considerably more precision than their Greco-Roman predecessors, the Hippocratic physician’s admonition, born from experience, remains true today. A physician’s obligation, then and now, lies in seeing, hearing, and treating the individual patient in front of her, in recognizing that person’s idiosyncrasies of nature and nurture and then reconciling them with general principles of medical knowledge.

Acknowledging the antiquity of debates about the tension between the general and the particular in medical practice, this paper examines how this issue has been addressed in the modern world. Shifts in perspective about experimental design—the movement toward randomized controlled studies, and the more recent interest in developing statistical models to leverage data gained from single case designs—may be understood as responses to this fundamental problem of moving between the particular and the general. Subsequent sections in this paper will explore the epistemological justification for and benefits of experimental designs that tend toward one end of this spectrum or the other, arguing—with reference to the antiquity of these debates—for the value of a co-existing balance between these two approaches to experimental design.

## 2. The Importance of Generalizability: Randomized Controlled Trials

In the decades following the Second World War, Henry Beecher, prompted largely by ethical abuses in human experimentation, sought to correct the shoddy execution of human studies research by identifying problematic experiments that had been recently conducted in the United States, many at respected academic institutions [10]. Much of his research and proposed guidelines focused on informed consent of study participants, though he also discussed the ethical challenges that can arise even when informed consent is obtained. Describing two studies investigating the benefits of thymectomy in myasthenia gravis, Beecher stressed that, even in these controlled studies, in which all participants had been informed that they could receive either the sham or the true surgical procedure, the researchers’ poor planning led them to expose participants to risks in order to test a surgery whose purported efficacy was rapidly disproven. The spectacular failure of thymectomy as a treatment for myasthenia gravis, Beecher argued, demonstrated “the necessity for sound planning in the clinic” and in conducting human studies research [11].

Yet Beecher was also aware of how difficult study design and interpretation could be. In “Experimentation on Man,” he highlights the problematic language and expectations of two of the Nuremberg Rules about physician behavior, which emerged from the trials of Nazi war criminals [12]:

Nuremberg Rule 2: The experiment should be such as to yield fruitful results for the good of society, unprocurable by other methods or means of study, and not random and unnecessary in nature.

Nuremberg Rule 3: The experiment should be so designed and based on the results of animal experimentation and a knowledge of the natural history of the disease or other problem under study that the anticipated results will justify the performance of the experiment.

Beecher responds to these well-intentioned Rules by questioning the predictability of scientific investigation. Many important discoveries have occurred by chance, he notes, and no researcher can be positively certain of the outcome of her research. Would an investigator necessarily know at the outset what is “random” or “unnecessary”? Emphasizing the challenges of using human subjects research to generate results that are predictably beneficial to many, he cautions [12]:

“But if the anticipated results fail to justify the performance of the experiment, has the investigator necessarily been guilty of wrong behavior? Who can guarantee the success of any new experiment? The aforementioned comments are by no means intended to scoff at this valiant effort to codify permissible experimentation in man. They are intended, rather, to indicate more clearly … how difficult it is to be precise in this field.”

The ensuing decades saw extensive governmental and institutional efforts to heed the call of Beecher and many others to adhere to ethical, consistent standards for the practice of human studies research, however problematic those standards might be in practice. The National Research Act (1974) and the Belmont Report (1979) began the process of establishing regulatory procedures for human research that were designed to mitigate dangerous imprecisions, to define what was considered random and unnecessary, to provide for the safety of study participants, and to ensure that studies would produce results that would benefit the lives of many more individuals than only those involved in the study. The US Food and Drug Administration (FDA) in particular has, over the course of the last 50 years, developed some of the most stringent drug testing policies in the world, and increasing amounts of safety and efficacy data are required to pass FDA regulations [13]. The justification for these efforts is the protection of the subjects involved in experiments and trials, as well as the protection of those who, though not part of a trial, would use the medications and procedures that have passed the rigorous trial process.

The value of research data for the purposes of meeting regulatory standards is determined in part by its place within the so-called “hierarchy of evidence.” Developed in the 1990s, this initial model of evaluating the utility and applicability of evidence privileges studies that include more people and allow for greater degrees of control over the kinds of evidence that are generated. The hierarchy, as described by Guyatt and summarized by Greenhalgh, is as follows [14,15]:

(1) Systematic reviews and meta­analyses
(2) Randomised controlled trials with definitive results (confidence intervals that do not overlap the threshold clinically significant effect)
(3) Randomised controlled trials with non­definitive results (a point estimate that suggests a clinically significant effect but with confidence intervals overlapping the threshold for this effect)
(4) Cohort studies
(5) Case­control studies
(6) Cross sectional surveys
(7) Case reports

One consequence of this method of according value to research data is the privileging of randomized controlled trials as a means of evaluating the efficacy and side effects of new drugs. These trials require large and relatively homogenous study populations in order to control for confounding variables. By some estimates, clinical trials involve less than 10% of individuals who suffer from the problem being investigated, because stringent inclusion and exclusion criteria, designed to facilitate the attribution of outcomes to the effect of the drug alone, are imposed on study enrollment procedures [16]. For drugs that are chemically similar but not identical to existing pharmacotherapies, especially large trials are needed to achieve the statistical significance necessary to demonstrate the benefit of the new drug over its predecessor [13]. This need for a large, relatively homogenous study population (a large N) has been increasingly difficult to meet in United States in recent years, as the American population has been variably exposed to more and more therapeutic interventions over its lifetime. Finding homogeneity is difficult in an ever more intervened-upon society. The evidentiary premium placed on acquiring a large, “treatment-naïve” study population has had a startling consequence: to meet statistical demands, imposed in part to ensure that the results are generalizable to many, more and more clinical trials are conducted outside of the United States, in low and middle income countries such as China and India. One study found that the top twenty American pharmaceutical companies conduct one third of their clinical trials outside of the United States [17].

The movement of clinical trials from the United States to low and middle income countries with treatment-naïve populations has occurred for multiple reasons and raises many ethical questions, but one question in particular is prominent in a consideration of the generalizability of large, randomized controlled trials: how widely applicable are results acquired from such assiduously selected study populations? In writing about ethical human experimentation, Beecher emphasized the importance of careful selection of study subjects [12]: they must be capable of consenting, and an investigator should be aware of how the patient’s physiological state at the time of the experiment might influence the results. The risk of confounding variables should be minimized so that the results of the therapeutic intervention can be clearly identified and measured. In keeping with this approach, a pharmaceutical company looking to test a drug in a large population can therefore control for the potential confounding effects of other medications by choosing a study population that never had the opportunity to be exposed to such other drugs. Countries like China and India—as well as others in South America, Africa, and southeast Asia—can easily meet these criteria. A great volume of data for a large, well-controlled N can be reliably generated [13]. Yet this desire to achieve the large, carefully-controlled N, with the goal of obtaining statistically robust results that are considered more relevant or generalizable, leads to at least one problematic feature about the relationship between the study population and the wider world. A large N, while conferring statistical power, does not necessarily guarantee generalizability, and a treatment-naïve population who lives in one circumscribed geographical location is, by design, different from many other populations who might seek to use the therapy being tested.

Even for drugs tested in the United States and Europe, attainment of therapeutic benefit in a large trial does not translate to similar efficacy in the general population [18]. Using number-needed-to-treat data about the top 10 highest grossing drugs in the United States, for example, Schork estimates that, for every one person they help, these medications fail to improve the health of between 3 and 24 people [19]. The assumption of the generalizability of the results of “gold standard” clinical trials is one reason why these drugs are prescribed, even if other data suggest that many patients taking these medications receive little to no benefit from them. Another study of antiretroviral drugs in HIV patients in the UK revealed that two large randomized controlled trials showed an unanticipated lack of generalizability to the broader community of individuals with HIV. The authors concluded that a probable reason for this disjunction between the study population and the general population involved the inability of the trials to account adequately for individual variability: in this context, differences in the way patients had acquired the HIV infection were predictive of one-year treatment follow-up rates. People who had acquired HIV through IV drug use or through heterosexual sexual contact were relatively under-represented in the study population, which consisted mostly of men who had acquired HIV through sex with men. As a result, when the conclusions of the study population were applied to the broader population of patients with HIV, the outcome was not as positive as expected, and more individuals than anticipated were lost to follow-up [20].

As these examples indicate, while large observational studies and extensive randomized controlled trials do yield demonstrably useful results in safe, regulated ways, their focus on achieving homogeneity for the sake of clear analysis of efficacy necessarily limits their generalizability. Although a modern experimental phenomenon that should rightly be celebrated as a scientific triumph, randomized controlled trials are not immune to the paradox of the particular and the general that the author of the Hippocratic *Places in Man* described so many centuries ago.

## 3. The Importance of Individuality: N-of-1 Trials and Single Case Designs

While randomized controlled trials and large observational studies celebrate the power of the standardized aggregate, precision medicine bases its claims to efficacy in the exhaustive collection of data about a single individual, A product of our knowledge of the human genome and the analytical possibilities afforded by ‘big data,’ precision medicine has reshaped ideas about the best treatment for a particular patient by focusing on the genetic code—its protective potentials, and its pitfalls—of an individual patient. In 2015, President Obama officially launched the Precision Medicine Initiative, which “will enable health care providers to tailor treatment and prevention strategies to people’s unique characteristics, including their genome sequence, microbiome composition, health history, lifestyle, and diet” [21,22]. By paying attention to such individual particulars, it is hoped, we can choose therapies that are uniquely suited to an individual’s genetic makeup and environmental exposures [19]. The ambition of precision medicine is to address the concerns recorded 2500 years ago by the author of *Places in Man*: precision medicine means that we can deal productively with individual variability, aligning diagnosis and treatment with the minute particulars of the singular patient. The collection of vast quantities of information, coupled with big data analytics, theoretically makes such customized treatments possible. A simultaneously exciting and troubling aspect of precision medicine, however, is that it defines itself—and even prides itself—on the principle of the N-of-1. This assumption about the utility of collecting and analyzing all available factors that influence the health of every individual, everywhere, undergirds arguments about the efficacy of treating the N-of-1. If analytics can identify all possibilities and outcomes with reasonable statistical certainty, then inputting millions or more of individual pieces of information about an individual into a database alongside similar metrics recorded about other people can lead a clinician down an algorithmic pathway to the therapeutic option that is specific for that particular patient [23]. Greater characterization of the individual, it is argued, means greater efficacy [19].

The possibility for specificity to the level of the individual genome and particular daily habits has had the surprising outcome of pivoting medical practice away from generalized theories and toward the idiosyncrasies of an individual’s DNA and lifestyle—toward therapeutic combinations that may have never been used before in those particular circumstances. Although ‘off-label’ use of therapeutics outside the data generated from large trials is not new [24], precision medicine provides innovative ways to search for and to justify the unique application of novel and developing treatments. The individuality of the patient is at the heart of such justification, but the idea of treating the individual with a therapy that has not been tested systematically before in humans seems on the surface to violate the key principles of evidence-based medicine. In their article discussing how evidence-based medicine and precision medicine might productively co-exist, Beckmann and Lew define evidence-based medicine as: “The use of evidence from well-designed and well-conducted research (such as from meta-analyses, systematic reviews, and randomized controlled trials) to optimize decision-making in medicine” [25]. The examples that they provide to illustrate the meaning of “well-designed and well-conducted” are those trials and studies at the top of the hierarchy of evidence. What role could single case designs and N-of-1 studies have in this understanding of evidence-based medicine?

Although N-of-1 trials, single case designs, and the concept of precision medicine focus on the individual, there are important areas of overlap and difference among these entities. Precision medicine, as described above, focuses on an individual’s genetic makeup. As intervention at the genetic level, these methodologies are typically irreversible. N-of-1 studies, N-of-1 trials, and single case designs, while focused on the individual, are not necessarily as tied to the concept of genetic intervention as precision medicine. Broadly, the term single case design (SCD) describes an experimental structure in which the subject serves as his or her own control. SCDs are useful because they can capture individual differences that might otherwise be hidden in group averages, but they can be difficult to analyze statistically. Recent endeavors by Shadish [26], among others, have provided new insight into how the data acquired from SCD studies might be effectively analyzed, thereby encouraging their increased use.

N-of-1 studies and N-of-1 trials are types of SCDs. Typically, N-of-1 trials are multi-crossover randomized trials in which a participant receives a (usually personalized) treatment multiple times. Such trials are often used to treat individuals with chronic conditions, since they afford the possibility of multiple crossovers between and among treatments [27], and they are useful in determining and characterizing the heterogeneity of treatment effects [27,28,29,30]. In a certain sense, however, the idea of an N-of-1 trial seems to run contrary to decades of careful standardization of human subjects research to ensure the safety of participants. The U.S. Department of Health and Human Services’ Office of Research Protection has defined research as: “a systematic investigation, including research development, testing and evaluation, designed to develop or contribute to generalizable knowledge…” [31]. By this definition, it could be possible to exclude N-of-1 trials from investigations classified as “research,” since their specificity could theoretically preclude contribution to “generalizable knowledge.” Yet although the traditional hierarchy of evidence accords the least value to research focused on single individuals, there are many reasons why N-of-1 trials should be accorded more value by clinicians, if we believe that the goal of research should be the provision of insights that can improve society, that is, the lives of the individuals who exist within it.

N-of-1 trials provide a number of important benefits to the patients and clinicians who are involved with their care. On the level of the relationship between the clinician and the patient, N-of-1 trials increase patients’ awareness of their condition and provide them with a greater sense of agency and control in matters of their own health. Since high levels of patient engagement correlate with improved outcomes [32], it is not unreasonable to conclude that the kind of discussions between clinicians and patients that occur in the context of N-of-1 trials could positively influence outcomes. Moreover, the utility of data acquired from the individual has gained new currency as precision medicine, genomics, and big data analytics play an increasing role in clinical practice [33]. Though their design can (although not necessarily) depart from that associated with randomized controlled trials, they can still provide the kind of statistical power afforded by such ‘gold standard’ trials through data aggregation. Purpose-built N-of-1 trial online platforms, for example, allow patients to input their own data in a standardized format that allows for individual choice and variation, yet also ensures the kind of consistency in data recording that enables statistical analysis [16].

Computational tools now exist to allow data from N-of-1 trials to be aggregated into a model that, unlike traditional randomized controlled trials and large observational studies, does not submerge or expunge individuality for the sake of achieving statistically clear results. These new models are better equipped to cope with statistical ‘noise’ and to make sense of it on both individual and population levels. This aggregated approach to N-of-1 trials is especially relevant for individuals with rarer pathological processes who are receiving experimental therapies that are impossible to test on a large scale [34]. Storage of data about particular patients allows for the accumulation of cases over time, which can then be compared to one another using analytical tools that are ever-increasing in their sophistication [35]. The active acquisition of N-of-1 trial data, coupled with its rigorous analysis, can and should have a place alongside data obtained from randomized controlled trials and large observational studies. While the latter two types of studies are excellent at demonstrating that new treatments have therapeutic benefit and a high level of internal validity, their very structure leads to inherent imprecisions in identifying precisely who will benefit, and how these individuals will benefit. Aggregated N-of-1 trial data can provide a way to enhance the external validity of treatment data obtained in these larger “gold standard” studies. The power and potential of these kinds of N-of-1 studies and trials is reflected in the 2011 revision made to the guidelines published by the Oxford Centre for Evidence-Based Medicine (OCEBM), which create space for randomized N-of-1 trials to be categorized as Level 1 evidence [36,37].

## 4. Moving between the Particular and the General: New Possibilities

As Jones, Grady, and Lederer note in an article celebrating the 50th anniversary of the publication of Beecher’s “Ethical and Clinical Research”: “There is not always consensus on what counts as ethical research, or who can be appropriate research subjects” [10]. Rules regarding the selection of “appropriate research subjects” for clinical trials were instituted both to protect individuals in those trials and to ensure that the results would be broadly applicable to the general population. However, as the preceding discussion has sought to show, generalizability can be surprisingly difficult to achieve and is not necessarily guaranteed by a randomized controlled trial. We face the same problem that the Hippocratic physicians articulated, a problem that provoked physicians of the subsequent centuries to develop variable and much-debated epistemological frameworks in an attempted to solve it: the tension between the particular and the general, the challenging of extracting general medical principles from individual encounters, and then applying that generalized knowledge in the setting of new encounters with different individuals. Today, however, the potential exists to examine individual variability in a way that generates hundreds of thousands, potentially millions, of data points for that single individual: these can then be added to a database of similar constellations of data from other individuals. Such management and curation of variables and individual variability affords the opportunity for prediction of outcomes based on similar alignment of variables, tailoring a diagnosis or a treatment to a patient in a way that aggregates others’ relevant data, with relevance determined through the algorithms that sift through individual and aggregate data. Since such transitions between particular and general are possible, SCDs and N-of-1 trials might be considered not only safer than a traditional clinical trial, but also more useful, provided that the treated individual’s data can then be transmitted back into the aggregated system.

Beecher’s concern with human experimentation emerged from the acknowledgement that the improvement of man’s condition depended upon such investigations [12]. Studies that enhance the quality and length of all human lives are therefore to be privileged, yet individual rights must at the same time be upheld and valued. In his words, “… the individual must not be subordinated to the community; the community exists for the man” [12]. As we move into an age of precision medicine, randomized controlled trials and large observational studies will still have their value, but we must question the extent to which they represent the subordination of the individual to the community, both in their execution and in their application. Equipped with new kinds of data and new ways to evaluate these data, physicians fall short in their duty to see their patients as individuals if they insist upon a blinkered imposition of the traditional hierarchy of evidence on the interpretation and application of scientific data. N-of-1 studies and trials provide an exciting new avenue for individuals to contribute to aggregated community data in ways that suit their own personal lives and goals, and big data analytics can provide a new means by which, to paraphrase Dr. Beecher, a community might exist for the individual.
